# Exercise Makes More than an Energy Deficit: Toward Improved Protocols for the Management of Obesity?

**DOI:** 10.1016/j.ebiom.2015.11.029

**Published:** 2015-11-18

**Authors:** Jean-Frederic Brun

**Affiliations:** PHYMEDEXP INSERM U1046, CNRS UMR 9214, University of Montpellier, France; Department of Clinical Physiology, Metabolic Exploration, University Hospital (CHU) of Montpellier, France

Iwayama's paper in *EBioMedicine* (Iwayama et al., 2015) may provide a significant advance in exercise-based strategies for the management of obesity and metabolic diseases. It demonstrates with a careful methodology that a bout of endurance exercise at moderate intensity (50% VO_2_max), when performed at fast, markedly increases 24 h fat oxidation. Although this finding is in accordance with some popular beliefs, it challenges the consensus of classical literature that concluded 10 years ago that exercise, despite a transient effect on the balance of substrate oxidation, was unable to significantly modify it over 24 h.

Classically, obesity was interpreted as a mismatch between energy intake and energy expenditure, so that restrictive diet was assumed to be its more logic treatment. A role for exercise was however suggested by studies on individuals engaged in vigorous physical activities, who are on the average leaner than sedentary people. Since 25 years it was thus well admitted that transforming an obese into an athlete could be a powerful treatment of obesity. Recently, an impressive evidence of this was provided by the RESOLVE study (Dutheil et al., 2013) that followed over 1 year obese subjects submitted to high volumes (15–20 h/wk) of various kinds of exercise (resistance and endurance at high vs low intensity). Whatever the protocol, high volumes of exercise had an impressive effect on weight and markers of metabolic syndrome. Unfortunately, all clinicians involved in the management of obesity have experienced that such a metamorphosis of obese into athletes, even if it is not impossible, can only be obtained in a quite reduced number of subjects and is not the most realistic solution for a majority of patients.

Over the last decade, the idea that exercise is an important component of obesity treatment has gained more credibility. In 2006 a meta-analysis concluded that exercise is crucial for avoiding weight gain, and stabilizing weight after diet-induced weight loss, but has a modest efficiency for weight loss (Bensimhon et al., 2006). However there was a growing evidence that exercise is slightly more efficient for decreasing weight than would be expected from energy deficit alone. A nice demonstration of this was provided by Ross (Ross et al., 2000) who showed that when exercise-induced energy expenditure was carefully compensated by food intake, there was still a weight loss, while the sedentary control group on the same period was gaining weight. The « study of a targeted risk reduction intervention through defined exercise » (STRRIDE) (Slentz et al., 2004) confirmed this finding and helped to understand how *exercise makes more than an energy deficit*.

The lessons of STRRIDE were that: (a) lack of exercise results in weight gain; (b) high volumes of exercise induce a marked weight loss with important metabolic improvements; (c) moderate volumes of exercise also induce weight loss but their effects on metabolism and body composition are not the same if exercise intensity is high or low. Low intensity exhibits specific effects on abdominal fat, lipid profile and insulin sensitivity.

Since high volumes of muscular activity are probably not a realistic solution for most patients, guidelines for physical activity in obesity were released, counseling to exercise more than 150 min/week (preferably 200–300 min/week). With such reduced volumes of exercise, it appears mandatory to optimize training protocols.

Actually most people believe that if exercise is of short duration it should be performed at a higher intensity in order to maximize its effects. This concept has led to a host of research on high intensity training that has strong effects on muscle biology but is not so easy to largely employ in obese patients, and whose long term effects on weight loss are not well known. In fact, low volumes of exercise at low intensity are at least as efficient on weight and glucoregulation than similar volumes at high intensity (Slentz et al., 2004). This finding is in line with a growing body of literature that proposes to target exercise at the levels were lipid oxidation is maximal, i.e. in the range 40–50% VO_2_max (Brun et al., 2012). Although low levels of such exercise involve little energy expenditure, they have been shown to be able to promote long lasting weight loss and a host of metabolic improvements (Besnier et al., 2015).

The concept of “metabolic program in skeletal muscle” (Houmard et al., 2011) provides an explanation of this paradox. In severe obesity and type 2 diabetes there are multiple molecular defects in muscle that contribute to reduced fat oxidation and persist when muscle cells are cultivated in vitro. This indicates that there is a dysfunctional metabolic program, which is presumably of an epigenetic origin. This program can be completely reversed by exercise training.

Therefore, exercise is more efficient in the management of obesity and diabetes than would be expected from energy deficit alone because it is a programmer of muscle metabolism.

In this context, will the finding of Iwayama and coworkers help us to better program muscle metabolism for decreasing fat stores on the long term? This remains to be investigated in follow-up studies. This study was performed on healthy young volunteers and it is not sure that in obese with impaired metabolic flexibility this mechanism is still working. In addition, the observed raise in lipid oxidation is explained by a shift in substrate balance due to glycogen depletion, a situation which generally increases appetite. Since weight loss in response to exercise is in part explained by a modification in eating behavior (King et al., 2008), it would be important to determine if a protocol based on exercise at fast actually decreases or increases subsequent food intake.

Interestingly, the authors show that the 24-hr increase in fat oxidation they observe is not only related to negative carbohydrate balance but also to negative fat balance. This may reflect another mechanism related to the effects of exercise at 50% VO_2_max, an intensity where muscle better uses fat as a fuel, on the ability to further oxidize more lipids. This has been largely studied in athletes, in whom training at fast shifts the balance of substrates toward more lipid oxidation (Marquet et al., 2015). In obese patients, training at this intensity also increases on the long term the ability of muscle to oxidize fat (Besnier et al., 2015, Brun et al., 2012), due to muscle metabolic adaptations that modify the abovementioned “metabolic program in skeletal muscle”.

On the whole, it seems clear that this paper further extents the interest of low to moderate intensity exercise (50% VO_2_max) which is easy to prescribe and already useful for reducing weight. It challenges the current notion that exercise does not modify the balance of substrate oxidation over 24 h, opening thus new perspectives for research. Whether this will result into improved exercise strategies in obesity remains to be studied.

## Disclosure

I declare no competing interests.
